# A cross-sectional study of Swiss ambulatory care services use by multimorbid patients in primary care in the light of the Andersen model

**DOI:** 10.1186/s12875-020-01221-x

**Published:** 2020-07-27

**Authors:** Mia Messi, Yolanda Mueller, Dagmar M. Haller, Andreas Zeller, Stefan Neuner-Jehle, Sven Streit, Bernard Burnand, Lilli Herzig

**Affiliations:** 1 Department of Family Medicine, Center for Primary Care and Public Health (Unisanté), University of Lausanne, Lausanne, Switzerland; 2 Primary Care Unit, Faculty of Medicine, University of Geneva, Geneva, Switzerland; 3 Center for Primary Health Care, University of Basel, Basel, Switzerland; 4 Institute of Primary Care, University of Zurich, Zürich, Switzerland; 5 Institute of Primary Health Care (BIHAM), University of Bern, Bern, Switzerland

**Keywords:** Primary care, Healthcare use, Multimorbidity

## Abstract

**Background:**

Multimorbidity is frequently encountered in primary care and is associated with increasing use of healthcare services. The Andersen Behavioral Model of Health Services Use is a multilevel framework classifying societal, contextual, and individual characteristics about the use of healthcare services into three categories: 1. predisposing factors, 2. enabling factors, and 3. need factors. The present study aimed to explore multimorbid patients’ use of ambulatory healthcare in terms of homecare and other allied health services, visits to GPs, and number of specialists involved. A secondary aim was to apply Andersen’s model to explore factors associated with this use.

**Method:**

In a cross-sectional study, 100 Swiss GPs enrolled up to 10 multimorbid patients each. After descriptive analyses, we tested the associations of each determinant and outcome variable of healthcare use, according to the Andersen model: predisposing factors (patient’s demographics), enabling factors (health literacy (HLS-EU-Q6), deprivation (DipCare)), and need factors (patient’s quality of life (EQ-5D-3L), treatment burden (TBQ), severity index (CIRS), number of chronic conditions, and of medications). Logistic regressions (dichotomous variables) and negative binomial regressions (count variables) were calculated to identify predictors of multimorbid patients’ healthcare use.

**Results:**

Analyses included 843 multimorbid patients; mean age 73.0 (SD 12.0), 28–98 years old; 48.3% men; 15.1% (127/843) used homecare. Social deprivation (OR 0.75, 95%CI 0.62–0.89) and absence of an informal caregiver (OR 0.50, 95%CI 0.28–0.88) were related to less homecare services use. The use of other allied health services (34.9% (294/843)) was associated with experiencing pain (OR 2.49, 95%CI 1.59–3.90). The number of contacts with a GP (median 11 (IQR 7–16)) was, among other factors, related to the absence of an informal caregiver (IRR 0.90, 95%CI 0.83–0.98). The number of specialists involved (mean 1.9 (SD 1.4)) was linked to the treatment burden (IRR 1.06, 95%CI 1.02–1.10).

**Conclusion:**

Multimorbid patients in primary care reported high use of ambulatory healthcare services variably associated with the Andersen model’s factors: healthcare use was associated with objective medical needs but also with contextual or individual predisposing or enabling factors. These findings emphasize the importance of adapting care coordination to individual patient profiles.

## Background

Multimorbidity is defined as the co-occurrence of two or more chronic medical conditions within one person [1–5]. Multimorbidity is well-known in primary care settings and is associated with increased healthcare use and costs [6]. This effect increases with the number of co-occurring chronic conditions [7–9]. Multimorbidity is associated with greater numbers of visits to general practitioners (GPs), specialist consultations, hospitalizations, and drug prescriptions [6, 7, 10–12]. In Germany, for example, multimorbid patients had more than twice as many contacts per year with physicians, and the number of consultations and the number of different physicians contacted increased steadily with each additional chronic condition [13].

The use of healthcare services is influenced by a complex system of societal, contextual, and individual factors. To explore these factors, researchers have developed explanatory models to help identify the most important predictors of healthcare services use [14]. Andersen’s Behavioral Model of Health Services Use provides a multilevel framework assigning those factors according to three categories: 1. predisposing factors, defined as demographic data and socioeconomic status, 2. enabling factors, defined by financial and organizational aspects, and 3. need factors, defined as the needs perceived by a patient and the needs evaluated by the GP (see also Methods section) [14]. Since the Andersen model was developed 50 years ago, it has evolved and integrated new variables [15, 16]. The model has been widely used by different disciplines, exploring different aspects of healthcare services use from societal, economic, philosophical, anthropological, structural, or medical points of views in different populations. The variables used in the model depend on the chosen perspective, and although they are not identical in each publication, they always refer to the three factors described above. A recent literature review showed that Andersen’s model is still commonly used as a theoretical framework for studies on a broad range of diseases and health service domains [17–19].

Little is known about the level of healthcare service use by multimorbid patients in primary care in Switzerland. The present study therefore aimed to explore multimorbid patients’ use of ambulatory healthcare services, including homecare and other allied health services, numbers of visits to GPs, and number of specialists consulted. A secondary aim was to apply Andersen’s model to explore the factors associated with healthcare services use in the studied population.

## Methods

### Study design and setting

Data for this study were extracted from the cross-sectional MultiMorbidity Study in Family Medicine (MMFM) conducted in Switzerland between January and September 2015. As the study’s detailed protocol and its first results have been described elsewhere [20, 21], we provide only a concise overview of the most important methodological details. In summary, a convenience sample of 100 GPs randomly enrolled 888 patients, aged 18 years and over, with at least three chronic conditions identified from a pre-established list of 75 conditions [22]. Missing values were deleted listwise, resulting in a final sample of 843 participants (94.9% of the initial sample). A minimum of three chronic conditions was preferred to enable an exploration of more complex situations. The profiles of participating GPs were similar in terms of age, sex, and practice location to those of Switzerland’s population of GPs as described in the Swiss Health Observatory’s Report *55* in 2016 [23]. Patients gave their written informed consent to participate.

GPs enrolled eligible patients using a personal pre-established random calendar. If an eligible patient refused to participate, then the GP documented their date of birth, sex, and reason for refusing [20]. GPs completed a written form for each patient enrolled, collecting the following variables to understand their healthcare services use in the 12 months preceding the index consultation: number of GP–patient contacts (including consultations, home visits, and telephone calls), number of medical specialists involved in the treatment (defined as other physicians seen by the patient), formal homecare services use, and use of other allied health services such as physiotherapy or occupational therapy. Patients also responded to a standardized telephone interview conducted by a trained research collaborator and including questions about the presence of an informal caregiver and measures of self-perceived treatment burden (TBQ score) [24], quality of life (EQ-5D-3L index) [25, 26], health literacy (HLS EU 6 score) [27, 28], and level of material, social and health deprivation (DipCare) [29].

The variables from patients’ and GPs’ questionnaire responses, which were potentially associated with the use of ambulatory healthcare services use, were divided into three categories (see Additional file 1) according to Andersen’s model [14, 30], as reviewed by de Boer [31] and Babtisch [17].

- **Predisposing factors** were defined as demographic data and socioeconomic status (e.g., age, sex, marital status).
- **Enabling factors** were defined as financial and organizational factors (e.g., deprivation score assessed using the DipCare Index [29], containing 16 questions about material, social, and health deprivation).
- **Need factors** included patients’ perceived health (self-evaluation of their own health status and needs according to the EQ-5D-3L index and the TBQ score) and health as evaluated by the GP (e.g., number of chronic conditions, number of medications, Cumulative Index Rating Scale (CIRS) score [32, 33], Severity Index (SI), and treatment burden).

One significant enabling factor usually considered is the health insurance coverage status. However, health insurance is mandatory for everyone living in Switzerland, and everybody has basic health and accident insurance which covers the costs of medical treatment and hospitalization [34]. All the study participants were considered to have basic coverage, at the very least, so this was not included as an enabling factor.

### Statistical analyses

After a descriptive analysis of the participants’ characteristics, we considered four different dependent variables: two binary (use of homecare services and use of other allied health services) and two count variables (number of contacts with a GP in the last 12 months and number of specialists involved). We tested associations between each factor and each dependent variable in a bivariate analysis using one-way ANOVAs, two-tailed *t*-tests, and Pearson’s chi-squared tests to compare differences in means and proportions, respectively. Variables that were associated (*p* <  0.20) were subsequently included in multivariate analyses. Logistic regressions (for dichotomous variables) and negative binomial regressions (for count variables) were calculated to identify the determinants of ambulatory healthcare use among multimorbid patients. We used negative binomial regression models because count-data models are more appropriate than standard linear regression models for non-negative integer-dependent variables and they fitted better than Poisson or zero-inflated modelling. We added the predisposing factors, enabling factors, and need factors into the regression model in a stepwise manner as independent variables, creating three models (see Additional File 1). Model 1 used predisposing factors alone, Model 2 integrated predisposing and enabling factors, and Model 3 was the complete model combining predisposing, enabling, and need factors (Table 2).

All analyses were performed using Stata software, version 14 (StataCorp LP, College Station, TX, USA). Missing values were deleted listwise, resulting in a final sample of 843 participants (94.9% of the initial sample).

#### Ethical approval

The present study was approved by the Human Research Ethics Committee of the Canton of Vaud (Protocol 315/14) on July 15, 2014.

## Results

### Descriptive analyses

Analyses included 843 patients from 28 to 98 years old, with a mean age of 73.0 (SD 12.0), 48.3% of whom were men. Their characteristics are reported in Table 1. The most frequent chronic conditions were hypertension, cardiovascular disease risk factors, diabetes, obesity, and ischemic heart disease, but 74/75 chronic conditions of the list were mentioned at least once.

**Table 1:** Socio-demographic and other characteristics of the multimorbid patient sample, *N* = 843

		All	Women	Men	*P*-value
**Participants**					
N (%)		843 (100)	436 (51.7)	407 (48.3)	–
**Age**					
Mean (SD)		73.0 (12.0)	72.9 (13.4)	73.1 (10.3)	0.848
Range (years)		28–98	28–97	33–98	
< 65 years, n (%)		187 (22.2)	107 (24.5)	80 (19.7)	*0.002*
65–74 years, n (%)		240 (28.5)	104 (23.8)	136 (33.4)	–
75–84 years, n (%)		278 (33.0)	140 (32.1)	138 (33.9)	–
≥ 85 years, n (%)		138 (16.4)	85 (19.5)	53 (13.0)	–
**Marital status**^c^					
Single, n (%)		80 (9.5)	50 (11.5)	30 (7.4)	*< 0.001*
Married, n (%)		418 (49.6)	155 (35.5)	263 (64.6)	–
Separated/Divorced, n (%)		143 (17.0)	75 (17.2)	68 (16.7)	–
Widowed, n (%)		202 (24.0)	156 (35.8)	46 (11.3)	–
**Mean number of adults in household**^c^ (SD)		1.7 (0.6)	1.5 (0.6)	1.8 (0.6)	*< 0.001*
**Presence of an informal caregiver**^c^, n (%)		572 (67.8)	292 (67.0)	280 (68.8)	0.571
**Educational level**^c^					
Primary, n (%)		186 (22.1)	124 (28.4)	62 (15.2)	*< 0.001*
Secondary, n (%)		319 (37.8)	181 (41.5)	138 (33.9)	–
Tertiary, n (%)		338 (40.1)	131 (30.0)	207 (50.9)	–
**Linguistic region**					
French-speaking, n (%)		325 (38.5)	173 (39.7)	152 (37.3)	0.487
German-speaking, n (%)		518 (61.4)	263 (60.3)	255 (62.6)	–
**Location of GP’s practice**					
Urban, n (%)		367 (43.5)	204 (46.8)	163 (40.0)	0.129
Semi-urban, n (%)		339 (40.2)	163 (37.4)	176 (43.2)	–
Rural, n (%)		137 (16.3)	69 (15.8)	68 (16.7)	–
**Mean number of chronic conditions** (SD)		5.4 (2.2)	5.4 (2.1)	5.5 (2.2)	0.519
**Mean number of medications** (SD)		7.7 (3.5)	7.9 (3.6)	7.5 (3.3)	0.067
**Mean CIRS score**^**a**^ (SD)		10.2 (4.3)	10.0 (4.3)	10.5 (4.3)	0.068
**Mean treatment burden** (evaluated by GP; SD)		4.5 (1.7)	4.6 (1.7)	4.4 (1.7)	0.109
**Mean TBQ score**^**b**, c^ (SD)		26.8 (18.8)	26.6 (18.6)	26.9 (19.0)	0.850
**DipCare Index**					
Mean material deprivation score (SD)		0.5 (1.3)	0.6 (1.4)	0.4 (1.1)	*0.014*
Mean social deprivation score (SD)		3.1 (1.4)	3.0 (1.4)	3.1 (1.4)	0.119
Mean healthcare deprivation score (SD)		0.5 (0.7)	0.5 (0.7)	0.4 (0.7)	*0.034*
**Quality of life (EQ-5D-3L)**^c^					
Mobility	Problematic, n (%)	372 (44.1)	210 (48.2)	162 (39.8)	*0.015*
Self-care	Problematic, n (%)	98 (11.6)	53 (12.2)	45 (11.1)	0.619
Usual activities	Problematic, n (%)	327 (38.8)	198 (45.4)	129 (31.7)	*< 0.001*
Pain/Discomfort	Problematic, n (%)	643 (76.3)	356 (81.6)	287 (70.5)	*< 0.001*
Anxiety/Depression	Problematic, n (%)	355 (42.1)	228 (52.3)	127 (31.2)	*< 0.001*
Visual Analog Scale (VAS), Mean (SD)		63.1 (19.4)	60.4 (19.4)	65.9 (19.0)	*< 0.001*

Results are expressed as the number of participants (percentage) or as an average ± standard deviation. *p-*values express differences between men and women

^a^*CIRS* Cumulative illness rating scale. ^b^*TBQ* Treatment burden questionnaire. ^c^Self-perceived

Details of the bivariate analyses involving the four dependent outcome variables (use of homecare services, use of other allied health services, number of contacts with GPs, and number of specialists involved) and the comparisons between the users and non-users of homecare and other allied health services can be found in the Supplementary Material (Additional file 1). Analyses of the intermediate Andersen models considering predisposing factors alone (Model 1) and predisposing and enabling factors together (Model 2) are in the Additional file 2.

Results from the multivariate analyses of the complete Andersen model (Model 3), integrating the predisposing, enabling, and need factors for our four outcomes are shown in Table 2. The final models revealed that the four outcomes were not equally associated with either the factors or the categories. Although the use of homecare services and the number of specialists consulted were associated with factors from all three categories (predisposing, enabling, and need), GP–patient contacts were associated with predisposing and need factors, and the use of other allied health services was only associated with need factors.

**Table 2:** Multivariate analysis of factors associated with the outcome variables of ambulatory healthcare services use (homecare, allied health services, number of GP–patient contacts and number of specialists consulted), considering Andersen’s model, controlled for predisposing factors + enabling factors + need factors; *N* = 843

			Homecare services	Allied health services	Contacts with GP in last 12 months	Number of specialists
			Odds ratio (95% CI)	Odds ratio (95% CI)	IRR (95% CI)	IRR (95% CI)
**Predisposing factors**						
**Sex**		Men	1 (ref.)	1 (ref.)	1 (ref.)	1 (ref.)
		Women	1.48 (0.88–2.49)	1.24 (0.91–1.69)	1.00 (0.92–1.08)	0.93 (0.84–1.04)
**Age**		< 65 years	1 (ref.)	1 (ref.)	1 (ref.)	1 (ref.)
		65–74 years	1.03 (0.43–2.50)	1.11 (0.69–1.77)	1.11 (0.99–1.25)	1.17 (1.00–1.37)
		75–84 years	1.82 (0.77–4.29)	0.78 (0.48–1.27)	1.21 (1.07–1.36)	1.19 (1.01–1.41)
		≥ 85 years	4.22 (1.62–10.99)	0.87 (0.49–1.53)	1.26 (1.08–1.46)	0.96 (0.77–1.20)
**Marital status**		Single	1 (ref.)	–	1 (ref.)	1 (ref.)
		Married	1.10 (0.38–3.18)	–	1.57 (1.35–1.82)	0.95 (0.77–1.17)
		Separated/Divorced	2.02 (0.88–4.65)	–	1.12 (0.99–1.26)	1.03 (0.87–1.21)
		Widowed	1.60 (0.73–3.54)	–	1.08 (0.95–1.23)	0.88 (0.74–1.05)
**Number of adults in household**			0.69 (0.38–1.25)	–	1.04 (0.96–1.12)	1.08 (0.97–1.20)
**Presence of an informal caregiver**		Presence	1 (ref.)	–	1 (ref.)	–
		Absence	0.50 (0.28–0.88)	–	0.90 (0.83–0.98)	–
**Educational level**		Primary	–	–	1 (ref.)	1 (ref.)
		Secondary	–	–	0.94 (0.85–1.04)	1.07 (0.93–1.24)
		Tertiary	–	–	0.86 (0.78–0.96)	1.21 (1.05–1.40)
**Linguistic region**		German-speaking	–	1 (ref.)	1 (ref.)	1 (ref.)
		French-speaking	–	0.80 (0.58–1.11)	0.80 (0.73–0.86)	1.23 (1.10–1.37)
**Location of GP’s practice**		Urban	–	–	–	1 (ref.)
		Semi-urban	–	–	–	0.88 (0.79–0.98)
		Rural	–	–	–	0.83 (0.72–0.97)
**Enabling factors**						
**Material deprivation**			0.85 (0.67–1.06)	1.07 (0.94–1.23)	–	1.00 (0.96–1.05)
**Social deprivation**			0.75 (0.62–0.89)	–	1.01 (0.98–1.04)	1.07 (1.03–1.12)
**Healthcare deprivation**			1.06 (0.70–1.61)	1.20 (0.91–1.57)	1.00 (0.93–1.07)	1.02 (0.93–1.11)
**Need factors**						
**Number of chronic conditions**			1.04 (0.93–1.16)	1.06 (0.98–1.15)	0.99 (0.97–1.01)	1.02 (1.00–1.05)
**Number of medications**			1.13 (1.05–1.21)	1.01 (0.96–1.06)	1.03 (1.02–1.04)	1.04 (1.02–1.06)
**CIRS score**^**a**^			1.00 (0.94–1.07)	1.01 (0.97–1.06)	1.01 (1.00–1.03)	1.01 (1.00–1.03)
**Treatment burden**			1.15 (0.98–1.33)	1.10 (0.99–1.22)	1.08 (1.05–1.10)	1.06 (1.02–1.10)
**TBQ score**^**b**^			1.00 (0.99–1.01)	1.00 (0.99–1.01)	1.00 (1.00–1.00)	1.00 (1.00–1.00)
**Perceived health state (EQ-5D-3L)**	Mobility	Non-problematic	1 (ref.)	1 (ref.)	1 (ref.)	1 (ref.)
		Problematic	1.26 (0.74–2.14)	1.27 (0.90–1.80)	0.99 (0.91–1.08)	1.01 (0.90–1.13)
	Self-care	Non-problematic	1 (ref.)	1 (ref.)	1 (ref.)	1 (ref.)
		Problematic	2.47 (1.36–4.51)	0.86 (0.52–1.42)	0.87 (0.77–0.99)	0.95 (0.80–1.12)
	Usual activities	Non-problematic	1(ref.)	1(ref.)	1(ref.)	1(ref.)
		Problematic	1.45 (0.84–2.49)	1.35 (0.93–1.94)	1.09 (1.00–1.20)	1.05 (0.92–1.18)
	Pain/Discomfort	Non-problematic	1 (ref.)	1 (ref.)	–	1 (ref.)
		Problematic	0.86 (0.45–1.66)	2.49 (1.59–3.90)	–	1.12 (0.98–1.28)
	Anxiety/ Depression	Non-problematic	1 (ref.)	–	1 (ref.)	–
		Problematic	1.18 (0.71–1.94)	–	1.04 (0.95–1.13)	–
	**Health scale (VAS)**		1.00 (0.98–1.01)	1.00 (0.99–1.01)	1.00 (1.00–1.00)	1.00 (1.00–1.00)
**Pseudo R**^**2**^			**0.27**	**0.09**	**0.04**	**0.07**

Statistical analyses conducted using logistic regressions for homecare services use and allied health services use and using negative binomial regressions for number of GP–patient contacts in the last 12 months and the number of specialist’s consulted, adjusting for all the variables indicated. ^a^*CIRS* Cumulative illness rating scale. ^b^*TBQ* Treatment burden questionnaire

### Homecare services

Homecare services were used by 15.1% (127/843) of the patient sample and use increased with age (> 85 years vs. < 65 years, OR 4.22, 95%CI 1.62–10.99). In the complete model, using a higher number of medications (OR 1.13, 95%CI 1.05–1.21) and being less independent (OR 2.47, 95%CI 1.36–4.51) were associated with the use of homecare services. It was noteworthy that social deprivation (OR 0.75, 95%CI 0.62–0.89) and the absence of an informal caregiver (OR 0.50, 95%CI 0.28–0.88) were, however, related to a lower homecare services use.

### Other allied health services

Other allied healthcare services (e.g. physiotherapy or occupational therapy) had been used by 34.9% (294/843) of patients during the preceding year. After adjusting for all factors (Model 3), only reporting pain or discomfort in the EQ-5D-3L questionnaire (OR 2.49, 95%CI 1.59–3.90) remained associated with the use of other allied health services.

### Contact with GPs

In the preceding 12 months, participants had a median of 11 contacts with their GP (IQR 7–16). Multivariate analysis showed that the predisposing factors of age (≥ 85 years vs. < 65 years, IRR 1.26, 95%CI 1.08–1.46) and the absence of an informal caregiver (IRR 0.90, 95%CI 0.83–0.98) were associated with the number of GP–patient contacts. Among need factors, a higher number of medications (IRR 1.03, 95%CI 1.02–1.04), a higher cumulative illness rating scale CIRS (IRR 1.01, 95%CI 1.00–1.03), a higher treatment burden (IRR 1.08, 95%CI 1.05–1.10) (as evaluated by the GP), and problems in performing usual activities of daily life (IRR 1.09, 95%CI 1.00–1.20) were associated with more GP–patient contacts.

### Number of specialists involved

Most patients, 84% (708/843), had consulted one or more specialists in the preceding 12 months, with a mean (SD) of 1.9 (1.4) specialists seen. There was no clear association between the number of specialists seen and age. The 75–84 years old age group was associated with a higher number of specialists consulted than those < 65 years old (IRR 1.19, 95%CI 1.01–1.41), but not higher than the oldest old (> 85 years, IRR 0.96, 95%CI 0.77–1.20). The fully adjusted model confirmed associations between a greater number of specialists consulted and certain predisposing factors, such as a higher educational level (tertiary education level, IRR 1.21, 95%CI 1.05–1.40) and linguistic region (higher in the French than the German-speaking region, IRR 1.23, 95%CI 1.10–1.37), and certain enabling factors, such as social deprivation (IRR 1.07, 95%CI 1.03–1.12). Need factors such as the number of chronic conditions (IRR 1.02, 95%CI 1.00–1.05), the number of medications prescribed (IRR 1.04, 95%CI 1.02–1.06), and the treatment burden (IRR 1.06, 95% 1.02–1.10) were also associated with the number of specialists consulted.

## Discussion

The present study analyzed the use of Swiss ambulatory healthcare services by multimorbid patients in primary care settings. Within the framework of Andersen Behavioral Model of Health Services Use, it explored the individual, contextual, and societal factors for four outcomes: the use of homecare services, the use of other allied health services, the numbers of GP–patient contacts, and the number of specialists involved. The factors associated with these four outcomes differed (e.g., use of other allied health services were associated with pain, whereas the number of GP–patient contacts was essentially associated with factors pertaining to medical severity). Interestingly, only 15% of the sample’s patients used homecare services. Andersen’s model revealed that need factors were associated with all four outcomes, predisposing factors with three (homecare, GP–patient contacts, and specialist consulted), and enabling factors with only two (homecare and specialists consulted). This shows that the use of ambulatory healthcare services depended, rationally, on objective medical needs, but also on contextual or individual factors.

As expected, multimorbid patients used more homecare services than the general population [7, 35], and greater age was significantly associated with higher use [36]. Despite a lack of social support usually being considered a predictor of healthcare services use [37], the present study found that social support seemed instead to be a promoter of its use. Indeed, patients with an informal caregiver or who were less socially deprived were more likely to use homecare services. These results are in line with those of a Canadian study by Lai L. et al. on Chinese immigrants [38].

Reporting pain or discomfort was the only factor associated with the use of other allied health services, which was the only outcome solely associated with need factors. This suggests that the use of other allied health services may be influenced solely by necessity. We found no previous study that specifically analyzed the use of other allied health services by multimorbid patients. Bähler et al. studied the costs linked using other allied health services, laboratory tests, and medical devices (“other outpatient costs”), whereas Heins et al. recorded that 46% of multimorbid patients without cancer used physical therapy and 7% used occupational therapy, which was in line with the present results [7, 39].

Multimorbid patients in the present study frequently consulted their GPs: this increased with age and the number and severity of their chronic conditions—a result in line with other European studies [13, 40]. However, Schellhorn et al. found that age did not play a significant role in predicting the frequency of visits to physicians by older Swiss adults [41]. GPs’ evaluations of need factors with regard to the number of GP–patient contacts showed an associations with the health status and the clinical severity of multimorbidity, confirming the results of previous studies [16, 42–44].

Interestingly, the present study revealed that although the number of specialists consulted initially increased with age, it decreased in the oldest old, in contrast to other studies [12, 45]. We could hypothesize that even though the oldest patients have increasing numbers of chronic conditions, they may have more difficulty attending specialist consultations or may set different priorities. Restricted access and discrimination are other factors potentially influencing the number of specialists consulted. Furthermore, the decreasing number of specialists consulted at older ages may be compensated by increasing contacts with GPs. This could suggest that their care tends towards a more holistic focus on the patients whole-person rather than on their specific diseases: close GP–patient relationships and the continuity of care prevent the overuse of specialist consultations [46]. Higher educational level has been described previously as a predictor of higher number of specialist consultations [45].

Our study found no associations between healthcare service use and disease or treatment burden as evaluated by the patients, which might have been thought of as obvious predictors of use. The concept of treatment burden is recent and there are few publications on this topic with none describes the specific relationship between treatment burden and healthcare services uses [47, 48].

The number of medications used was associated with three outcomes (homecare services, GP–patient contacts, and specialists consulted). Unsurprisingly, the number of medications used increased with the number of chronic conditions [8, 16, 43, 49].

As described above, Andersen’s model enabled us to analyze and classify individual, societal, and contextual characteristics according to the three categories of factors of ambulatory healthcare use: predisposing factors, enabling factors, and need factors. This model was initially designed as a tool to analyze inequalities in healthcare services use [17, 30, 31]. In the present study, predisposing and need factors contributed most to explain ambulatory healthcare services use. However, each ambulatory healthcare use outcome was associated with a different combination of factors, illustrating the complexity of caring for multimorbid patients when all three factors of the Andersen model can influence a patient’s healthcare services use. We suggest that this indicates the importance of GPs developing more patient-centered care rather than disease-oriented care as proposed by most guidelines. Indeed, May et al. proposed starting with “treatments for patients and not for diseases” [50]. This is in line with other authors’ suggestions of giving less importance to disease-centered care and starting to care for patients holistically [51, 52]. In reality, demographic and socioeconomic status or the individual’s perceived health may have more influence on healthcare services use than any given chronic condition. As multimorbity grows with population aging, caregivers and political decision-makers should be aware of the need to integrate all three factors from the Anderson model into planning healthcare access and thus reducing inequalities. Further research about ambulatory healthcare services use by multimorbid patients in primary care will be needed to better explain the associations revealed here, and this should involve integrating the variables and reflections of the Andersen model.

### Strengths and limitations

The present study’s strengths include its large population of multimorbid patients from primary care settings across the country, and its inclusion of all adults over 18 years old instead of focusing on older adults, as do most studies on multimorbidity. Furthermore, multimorbidity was defined as three or more of 75 chronic conditions on a pre-established list, thus giving a broader picture of multimorbidity’s complexity. Finally, the study assessed a significant range of social and medical variables and factors with potential associations to multimorbid patients’ medical and homecare use in primary care settings, thus helping to provide a more global picture of the situation in Switzerland.

Nevertheless, the present results should be considered in light of certain limitations. First, the cross-sectional design merely demonstrated associations, and causal relationships could not be inferred. Second, Andersen’s model is quite an old framework, developed 50 years ago, even if several researchers still used it recently [17–19, 30, 31]. The model has evolved over the years and has not been used with exactly the same variables in all publications, depending on the discipline using it. The present study included most of the variables in Andersen’s three factors, but some were not included in our concept (i.e., cultural beliefs or organizational factors). However, we found no other framework enabling an exploration of the economic, sociodemographic, or societal aspects of healthcare services use by multimorbid patients, and we think that the model added some very important information to our analyses. Combining multiple measures into a single multivariate model, especially the underlying *need factors*, may have over-adjusted the model and diluted the effects of specific measures. It is thus difficult to draw conclusions from a multivariate model containing every variable as this creates a major risk of collinearity. The sometimes-divergent results showed the complexity of the relationships between predisposing, enabling, and need factors, as well as the limitations of a theoretical model. Third, one important characteristic of the Swiss healthcare system is that GPs do not fulfil a gatekeeper function: patients are free to consult specialists directly without informing their GP, implying that the number of those consultations may have been underestimated.

Our findings suggest that new, more precise means of analyzing multimorbid patients’ use of healthcare services are needed. Further studies exploring the complex associations highlighted here should be conducted.

## Conclusion

The present study described the use of ambulatory healthcare services by multimorbid patients in a primary care setting in Switzerland. It revealed that their use was variably associated with the predisposing factors, enabling factors, and need factors described within the framework of the Andersen model. The care of multimorbid patients is therefore complex, and the population is far from homogenous. Our findings emphasized the importance of adapting care coordination to patients’ individual profiles and highlighted that GPs should increase their efforts to provide a patient-centered style of care, rather than the more traditional, disease-oriented care.

## Supplementary information

**Additional file 1.** Andersen Model.

**Additional file 2.** Utilization of ambulatory healthcare services.

## Data Availability

Data are available at the Department of Family Medicine, Center for Primary Care and Public Health (Unisanté), University of Lausanne, Switzerland.

## Abbreviations

GPs: General practitioners
MMFM: MultiMorbidity in Family Medicine
