# Supplementary material for: A cross-sectional study of Swiss ambulatory care services use by multimorbid patients in primary care in the light of the Andersen model
Source: BMC Fam Pract. 2020 Jul 27;21:150. doi: 10.1186/s12875-020-01221-x (PMC7385958; doi:10.1186/s12875-020-01221-x)
Supplement: Supplementary file 1 — Additional file 1. Andersen Model. [file 12875_2020_1221_MOESM1_ESM.docx]

Additional file (Andersen Model)

Model 1: controlled for predisposing factors

Model 2: controlled for predisposing factors (model 1) + enabling factors

Model 3: controlled for predisposing factors + enabling factors (model 2) + need factors

Figure 1. Adaptation of Andersen model

HEALTH BEHAVIOR

Personal health practices

Process of medical Care

Use of healthcare services

ENVIRONMENT CHARACTERISTICS

OUTCOME

Perceived health

Evaluated

health

Consumer satisfaction

PREDISPOSING FACTORS

Demographics

Social structure

Beliefs

ENABLING FACTORS

Health Policy

Financing

Organization

NEED FACTORS

Environmental

Population heath indices

ENABLING FACTORS

Financing

Organization

NEED FACTORS

Perceived health

Evaluated health

PREDISPOSING FACTORS

Demographics

Social structure

Beliefs

POPULATION CHARACTERISTICS

Figure 2. Diagram presenting Models 1, 2 and 3 used in the multivariate analysis.

Model 3

Model 2

Model 1
