# Supplementary material for: A cross-sectional study of Swiss ambulatory care services use by multimorbid patients in primary care in the light of the Andersen model
Source: BMC Fam Pract. 2020 Jul 27;21:150. doi: 10.1186/s12875-020-01221-x (PMC7385958; doi:10.1186/s12875-020-01221-x)
Supplement: Supplementary file 2 — Additional file 2. Utilization of ambulatory healthcare services. [file 12875_2020_1221_MOESM2_ESM.docx]

**Utilization of ambulatory healthcare services - Tables - Supplement**

Table A. Descriptive analyses and bivariate analysis of factors associated with ambulatory care services utilization, N=843.

*Numerical data are presented as mean and standard deviation,* categorical data as absolute value and percentage.

|  | | | | | | | **Outcomes** | |  |  |  |  |  |  |  |  |  |  |  |  |
| --- | --- | --- | --- | --- | --- | --- | --- | --- | --- | --- | --- | --- | --- | --- | --- | --- | --- | --- | --- | --- |
|  | | | | | | | **Homecare services** | | | | **Paramedical involvement** | | | | **Contacts with GP in 12 months** | | | **Number of specialists** | | |
|  | | | | | | | **Yes**  N (%)  *Mean (SD)* | **No**  N (%)  *Mean (SD)* | ***p-*value***** | ***p*-value*^+^** | **Yes**  N (%)  *Mean (SD)* | **No**  N (%)  *Mean (SD)* | ***p-*value***** | ***p*-value*^+^** | **Mean (SD)** | ***p-*value***** | ***p*-value*^+^** | **Mean (SD)** | ***p-*value***** | ***p*-value*^+^** |
| **Predisposing factors** | | | | | | |  |  |  |  |  |  |  |  |  |  |  |  |  |  |
|  | **Sexe** | | | Men | | | 42 (10.3) | 365 (89.7) | **<0.001** | **-** | 124 (30.5) | 283 (69.5) | **0.009** | **-** | 12.3 (8.2) | **0.031** | **-** | 2.0 (1.5) | **0.023** | **-** |
|  |  | | | Women | | | 85 (19.5) | 351 (80.5) |  |  | 170 (39.0) | 266 (61.0) |  |  | 13.6 (9.3) |  |  | 1.8 (1.5) |  |  |
|  | **Age** | | | <65 years | | | 16 (8.6) | 171 (91.4) | **<0.001** | **-** | 74 (39.6) | 113 (60.4) | 0.399 | - | 12.2 (9.9) | **0.030** | **-** | 1.9 (1.4) | **<0.001** | **-** |
|  |  | | | 65-74 years | | | 17 (7.1) | 223 (92.9) |  |  | 85 (35.4) | 155 (64.6) |  |  | 11.9 (8.3) |  |  | 2.0 (1.5) |  |  |
|  |  | | | 75-84 years | | | 48 (17.3) | 230 (82.7) |  |  | 91 (32.7) | 187 (67.3) |  |  | 13.9 (8.7) |  |  | 2.1 (1.5) |  |  |
|  |  | | | 85+ years | | | 46 (33.3) | 92 (66.7) |  |  | 44 (31.9) | 94 (68.1) |  |  | 13.7 (7.9) |  |  | 1.4 (1.3) |  |  |
|  | **Marital status** | | | Single | | | 9 (11.2) | 71 (88.7) | **<0.001** | **0.007** | 29 (36.2) | 51 (63.7) | 0.063 | 0.741 | 17.3 (15.0) | **<0.001** | 0.091 | 1.7 (1.2) | **<0.001** | 0.168 |
|  |  | | | Married | | | 37 (8.8) | 381 (91.1) |  |  | 140 (33.5) | 278 (66.5) |  |  | 11.9 (7.1) |  |  | 2.1 (1.6) |  |  |
|  |  | | | Separated / Divorced | | | 24 (16.8) | 119 (83.2) |  |  | 63 (44.1) | 80 (55.9) |  |  | 12.8 (8.4) |  |  | 2.1 (1.3) |  |  |
|  |  | | | Widow(er) | | | 57 (28.2) | 145 (71.8) |  |  | 62 (30.7) | 140 (69.3) |  |  | 13.6 (8.4) |  |  | 1.6 (1.4) |  |  |
|  | ***Number of adults in household, mean (SD)*** | | | | | | *1.4 (0.6)* | *1.7 (0.6)* | ***<0.001*** | ***0.001*** | *1.6 (0.7)* | *1.7 (0.6)* | *0.435* | *0.483* | ***-*** | ***0.002*** | ***0.022*** | ***-*** | ***0.003*** | ***0.033*** |
|  | **Presence of informal caregiver** | | | | | Yes | 106 (18.5) | 466 (81.5) | **<0.001** | **0.001** | 201 (35.1) | 371 (64.9) | 0.815 | 0.619 | 13.4 (8.9) | **0.025** | **0.015** | 1.9 (1.5) | 0.567 | 0.482 |
|  |  | | | | | No | 21 (7.7) | 250 (92.2) |  |  | 93 (34.3) | 178 (65.7) |  |  | 11.9 (8.3) |  |  | 1.9 (1.5) |  |  |
|  | **Schooling level** | | | | | Primary | 33 (17.7) | 153 (82.3) | 0.326 | 0.927 | 62 (33.3) | 124 (66.7) | 0.738 | 0.224 | 13.4 (8.9) | 0.123 | 0.124 | 1.7 (1.5) | **0.002** | **0.002** |
|  |  | | | | | Secondary | 50 (15.7) | 269 (84.3) |  |  | 109 (34.2) | 210 (65.8) |  |  | 13.4 (9.4) |  |  | 1.8 (1.4) |  |  |
|  |  | | | | | Tertiary | 44 (13.0) | 294 (86.9) |  |  | 123 (36.4) | 215 (63.6) |  |  | 12.2 (8.1) |  |  | 2.1 (1.5) |  |  |
|  | **Linguistic region** | | | | | German | 83 (16.0) | 435 (84.0) | 0.326 | 0.376 | 191 (36.9) | 327 (63.1) | 0.125 | 0.084 | 14.2 (9.7) | **<0.001** | **<0.001** | 2.0 (1.4) | 0.128 | 0.137 |
|  |  | | | | | French | 44 (13.5) | 281 (86.5) |  |  | 103 (31.7) | 222 (68.3) |  |  | 11.0 (6.5) |  |  | 1.9 (1.5) |  |  |
|  | **Localization of GP’s office** | | | | | Urban | 57 (15.5) | 310 (84.5) | 0.919 | 0.780 | 133 (36.2) | 234 (63.8) | 0.677 | 0.447 | 13.1 (8.6) | 0.822 | 0.999 | 2.0 (1.5) | 0.318 | 0.105 |
|  |  | | | | | Semi-urban | 49 (14.4) | 290 (85.5) |  |  | 117 (34.5) | 222 (65.5) |  |  | 12.7 (8.7) |  |  | 1.9 (1.5) |  |  |
|  |  | | | | | Rural | 21 (15.1) | 116 (84.7) |  |  | 44 (32.1) | 93 (67.9) |  |  | 13.1 (9.4) |  |  | 1.8 (1.4) |  |  |
| **Enabling factors** | | | | | | |  |  |  |  |  |  |  |  |  |  |  |  |  |  |
|  | | ***DipCare index*** | | | | | *1.3 (0.9)* | *1.6 (0.9)* | ***<0.001*** | ***0.152*** | *1.7 (1.0)* | *1.5 (0.8)* | ***0.001*** | ***0.015*** | *-* | *0.233* | *0.353* | *-* | ***0.002*** | ***0.008*** |
|  | | ***Material Deprivation*** | | | | | *0.5 (1.0)* | *0.5 (1.3)* | *0.794* | ***0.010*** | *0.7 (1.5)* | *0.4 (1.1)* | ***<0.001*** | ***0.008*** | *-* | *0.408* | *0.206* | *-* | *0.057* | *0.141* |
|  | | ***Social Deprivation*** | | | | | *2.3 (1.1)* | *3.2 (1.4)* | ***<0.001*** | ***<0.001*** | *3.0 (1.4)* | *3.1 (1.4)* | *0.451* | *0.299* | *-* | ***<0.001*** | ***0.001*** | *-* | *0.073* | *0.171* |
|  | | ***Healthcare Deprivation*** | | | | | *0.7 (0.7)* | *0.4 (0.7)* | ***<0.001*** | ***<0.001*** | *0.7 (0.7)* | *0.4 (0.6)* | ***<0.001*** | ***<0.001*** | *-* | ***<0.001*** | ***0.001*** | *-* | ***<0.001*** | ***<0.001*** |
| **Need factors** | | | | | | |  |  |  |  |  |  |  |  |  |  |  |  |  |  |
|  | | ***Number of chronic conditions*** | | | | | *6.3 (2.9)* | *5.2 (2.0)* | ***<0.001*** | ***<0.001*** | *5.8 (2.4)* | *5.2 (2.0)* | ***<0.001*** | ***<0.001*** | *-* | ***<0.001*** | ***<0.001*** | *-* | ***<0.001*** | ***<0.001*** |
|  | | ***Number of medications*** | | | | | *9.8 (4.0)* | *7.3 (3.3)* | ***<0.001*** | ***<0.001*** | *8.4 (3.8)* | *7.3 (3.3)* | ***<0.001*** | ***<0.001*** | *-* | ***<0.001*** | ***<0.001*** | *-* | ***<0.001*** | ***<0.001*** |
|  | | ***CIRS score°*** | | | | | *12.2 (4.7)* | *9.9 (4.2)* | ***<0.001*** | ***<0.001*** | *11.1 (4.5)* | *9.8 (4.2)* | ***<0.001*** | ***<0.001*** | *-* | ***<0.001*** | ***<0.001*** | *-* | ***<0.001*** | ***<0.001*** |
|  | | ***Burden of treatment*** | | | | | *5.3 (1.7)* | *4.4 (1.7)* | ***<0.001*** | ***<0.001*** | *5.0 (1.7)* | *4.3 (1.7)* | ***<0.001*** | ***<0.001*** | *-* | ***<0.001*** | ***<0.001*** | *-* | ***<0.001*** | ***<0.001*** |
|  | | ***TBQ score°°*** | | | | | *28.2 (21.6)* | *26.5 (18.2)* | *0.355* | ***<0.001*** | *29.6 (20.3)* | *25.2 (17.8)* | ***0.001*** | ***0.009*** | *-* | *0.093* | ***0.018*** | *-* | ***<0.001*** | ***<0.001*** |
|  | | **Perceived health state (EQ-5D-3L)** | Mobility | | Not problematic | | 40 (8.5) | 431 (91.5) | **<0.001** | **<0.001** | 128 (27.2) | 343 (72.8) | **<0.001** | **<0.001** | 11.9 (8.6) | **<0.001** | **<0.001** | 1.8 (1.3) | **0.005** | **0.001** |
|  | |  |  |  | Problematic | | 87 (23.4) | 285 (76.6) |  |  | 166 (44.6) | 206 (55.4) |  |  | 14.3 (8.8) |  |  | 2.1 (1.6) |  |  |
|  | |  | Self-care | | Not problematic | | 86 (11.5) | 659 (88.5) | **<0.001** | **<0.001** | 248 (33.3) | 497 (66.7) | **0.008** | **0.007** | 12.8 (8.9) | 0.174 | 0.163 | 1.9 (1.4) | 0.056 | **0.045** |
|  | |  |  |  | Problematic | | 41 (41.8) | 57 (58.2) |  |  | 46 (46.9) | 52 (53.1) |  |  | 14.1 (7.8) |  |  | 2.2 (1.7) |  |  |
|  | |  | Usual activities | | Not problematic | | 48 (9.3) | 468 (90.7) | **<0.001** | **<0.001** | 140 (27.1) | 376 (72.9) | **<0.001** | **<0.001** | 11.9 (8.4) | **<0.001** | **<0.001** | 1.8 (1.3) | **<0.001** | **<0.001** |
|  | |  |  |  | Problematic | | 79 (24.2) | 248 (75.8) |  |  | 154 (47.1) | 173 (52.9) |  |  | 14.6 (9.1) |  |  | 2.2 (1.6) |  |  |
|  | |  | Pain / Discomfort | | Not problematic | | 21 (10.5) | 179 (89.5) | **0.039** | 0.058 | 31 (15.5) | 169 (84.5) | **<0.001** | **<0.001** | 12.3 (10.2) | 0.238 | 0.287 | 1.6 (1.2) | **<0.001** | **<0.001** |
|  | |  |  |  | Problematic | | 106 (16.5) | 537 (83.5) |  |  | 263 (40.9) | 380 (59.1) |  |  | 13.1 (8.3) |  |  | 2.0 (1.5) |  |  |
|  | |  | Anxiety / Depression | | Not problematic | | 62 (12.7) | 426 (87.3) | **0.025** | **0.001** | 156 (32.0) | 332 (68.0) | **0.038** | 0.283 | 12.3 (8.4) | **0.020** | **0.010** | 1.9 (1.4) | 0.325 | 0.268 |
|  | |  |  |  | Problematic | | 65 (18.3) | 290 (81.7) |  |  | 138 (38.9) | 217 (61.1) |  |  | 13.8 (9.2) |  |  | 2.0 (1.4) |  |  |
|  | |  | ***Health scale (VAS)*** | | | | *56.2 (20.2)* | *64.3 (19.0)* | ***<0.001*** | ***<0.001*** | *58.2 (19.9)* | *65.6 (18.6)* | ***<0.001*** | ***<0.001*** | *-* | ***<0.001*** | ***<0.001*** | *-* | ***<0.001*** | ***<0.001*** |

**p-*value express statistical analysis with chi-square or by Student’s t-test for categorical variables and bivariate negative binomial regression for continuous variables. ^+^adjusted for age and gender

°Cumulative illness rating scale. °°Treatment burden questionnaire.

Table B. Multivariate analysis of factors associated with ambulatory healthcare services utilization, N=843

1. Logistic regression for homecare services use (Odds ratio (OR) and 95% confidence interval (95% CI))

|  | | | Model 1 | | Model 2 |  | Model 3 | |
| --- | --- | --- | --- | --- | --- | --- | --- | --- |
|  |  |  | Odds ratio (95% CI) | *p*-value | Odds ratio (95% CI) | *p*-value | Odds ratio (95% CI) | *p*-value |
| **Predisposing factors** | **Sex** | Men | 1 (ref.) |  | 1 (ref.) |  | 1 (ref.) |  |
|  |  | Women | 1.55 (0.99 – 2.42) | 0.057 | 1.62 (1.001 – 2.60) | **0.044** | 1.48 (0.88 – 2.49) | 0.137 |
|  | **Age** | <65 years | 1 (ref.) |  | 1 (ref.) |  | 1 (ref.) |  |
|  |  | 65-74 years | 0.95 (0.45 – 2.01) | 0.899 | 1.25 (0.55 – 2.82) | 0.599 | 1.03 (0.43 – 2.50) | 0.938 |
|  |  | 75-84 years | 2.07 (1.07 – 4.01) | **0.032** | 2.47 (1.14 – 5.37) | **0.022** | 1.82 (0.77 – 4.29) | 0.168 |
|  |  | 85+ years | 3.93 (1.88 – 8.24) | **<0.001** | 4.52 (1.90 – 10.76) | **0.001** | 4.22 (1.62 – 10.99) | **0.003** |
|  | **Martial state** | Married | 1 (ref.) |  | 1 (ref.) |  | 1 (ref.) |  |
|  |  | Single | 1.00 (0.38 – 2.62) | 0.993 | 0.87 (0.33 – 2.34) | 0.786 | 1.10 (0.38 – 3.18) | 0.860 |
|  |  | Separated / Divorced | 1.96 (0.94 – 4.11) | 0.074 | 1.76 (0.82 – 3.80) | 0.147 | 2.02 (0.88 – 4.65) | 0.097 |
|  |  | Widow(er) | 1.54 (0.74 – 3.18) | 0.247 | 1.53 (0.73 – 3.22) | 0.259 | 1.60 (0.73 – 3.54) | 0.243 |
|  | **Number of adults in the household** | | 0.62 (0.35 – 1.08) | 0.089 | 0.68 (0.39 – 1.18) | 0.170 | 0.69 (0.38 – 1.25) | 0.222 |
|  | **Informal caregiver** | Presence | 1 (ref.) |  | 1 (ref.) |  | 1 (ref.) |  |
|  |  | Absence | 0.37 (0.22 – 0.63) | **<0.001** | 0.38 (0.22 – 0.64) | **<0.001** | 0.50 (0.28 – 0.88) | **0.016** |
|  | **Schooling level** | Primary | - | - | - | - | - | - |
|  |  | Secondary | - | - | - | - | - | - |
|  |  | Tertiary | - | - | - | - | - | - |
|  | **Linguistic region** | German | - | - | - | - | - | - |
|  |  | French | - | - | - | - | - | - |
| **Enabling factors** | **Material Deprivation** | | - | - | 0.90 (0.73 – 1.10) | 0.308 | 0.85 (0.67 – 1.06) | 0.153 |
|  | **Social Deprivation** | | - | - | 0.69 (0.58 – 0.81) | **<0.001** | 0.75 (0.62 – 0.89) | **0.001** |
|  | **Healthcare Deprivation** | | - | - | 1.96 (1.43 – 2.71) | **<0.001** | 1.06 (0.70 – 1.61) | 0.772 |
| **Need factors** | **Number of chronic conditions** | | - | - | - | - | 1.04 (0.93 – 1.16) | 0.486 |
|  | **Number of medications** | | - | - | - | - | 1.13 (1.05 – 1.21) | **0.001** |
|  | **Severity index (CIRS)** | | - | - | - | - | 1.00 (0.94 – 1.07) | 0.931 |
|  | **Burden of treatment** | | - | - | - | - | 1.15 (0.98 – 1.33) | 0.077 |
|  | **TBQ score** | | - | - | - | - | 1.00 (0.99 – 1.01) | 0.956 |
|  | **Mobility** | Not problematic | - | - | - | - | 1 (ref.) |  |
|  |  | Problematic | - | - | - | - | 1.26 (0.74 – 2.14) | 0.383 |
|  | **Autonomy** | Not problematic | - | - | - | - | 1 (ref.) |  |
|  |  | Problematic | - | - | - | - | 2.47 (1.36 – 4.51) | **0.003** |
|  | **Usual activities** | Not problematic | - | - | - | - | 1(ref.) |  |
|  |  | Problematic | - | - | - | - | 1.45 (0.84 – 2.49) | 0.185 |
|  | **Pain / Discomfort** | Not problematic | - | - | - | - | 1 (ref.) |  |
|  |  | Problematic | - | - | - | - | 0.86 (0.45 – 1.66) | 0.656 |
|  | **Anxiety / Depression** | Not problematic | - | - | - | - | 1 (ref.) |  |
|  |  | Problematic | - | - | - | - | 1.18 (0.71 – 1.94) | 0.525 |
|  | **Health State (VAS)** | | - | - | - | - | 1.00 (0.98 – 1.01) | 0.651 |
|  | **Pseudo R^2^** | | **0.13** |  | **0.19** |  | **0.27** |  |

1. Logistic regression for other paramedical services use (Odds ratio (OR) and 95% confidence interval (95% CI))

|  | | | Model 1 | | Model 2 |  | Model 3 | |
| --- | --- | --- | --- | --- | --- | --- | --- | --- |
|  |  |  | Odds ratio (95% CI) | *p*-value | Odds ratio (95% CI) | *p*-value | Odds ratio (95% CI) | *p*-value |
| **Predisposing factors** | **Sex** | Men | 1 (ref.) |  | 1 (ref.) |  | 1 (ref.) |  |
|  |  | Women | 1.48 (1.11 – 1.97) | **0.008** | 1.40 (1.04 – 1.88) | **0.025** | 1.24 (0.91 – 1.69) | 0.178 |
|  | **Age** | <65 years | 1 (ref.) |  | 1 (ref.) |  | 1 (ref.) |  |
|  |  | 65-74 years | 0.86 (0.58 – 1.28) | 0.461 | 1.26 (0.81 – 1-96) | 0.309 | 1.11 (0.69 – 1.77) | 0.676 |
|  |  | 75-84 years | 0.74 (0.50 – 1.09) | 0.132 | 1.09 (0.70 – 1.69) | 0.699 | 0.78 (0.48 – 1.27) | 0.324 |
|  |  | 85+ years | 0.69 (0.43 – 1.10) | 0.122 | 1.10 (0.65 – 1.84) | 0.724 | 0.87 (0.49 – 1.53) | 0.629 |
|  | **Martial state** | Married | - | - | - | - | - | - |
|  |  | Single | - | - | - | - | - | - |
|  |  | Separated / Divorced | - | - | - | - | - | - |
|  |  | Widow(er) | - | - | - | - | - | - |
|  | **Number of adults in the household** | | - | - | - | - | - | - |
|  | **Informal caregiver** | Presence | - | - | - | - | - | - |
|  |  | Absence | - | - | - | - | - | - |
|  | **Schooling level** | Primary | - | - | - | - | - | - |
|  |  | Secondary | - | - | - | - | - | - |
|  |  | Tertiary | - | - | - | - | - | - |
|  | **Linguistic region** | German | 1 (ref.) |  | 1 (ref.) | - | 1 (ref.) |  |
|  |  | French | 0.77 (0.58 – 1.04) | 0.092 | 0.77 (0.57 – 1.04) | 0.085 | 0.80 (0.58 – 1.11) | 0.179 |
| **Enabling factors** | **Material Deprivation** | | - | - | 1.11 (0.97 – 1.26) | 0.123 | 1.07 (0.94 – 1.23) | 0.313 |
|  | **Social Deprivation** | | - | - | - | - | - | - |
|  | **Healthcare Deprivation** | | - | - | 1.69 (1.35 – 2.12) | **<0.001** | 1.20 (0.91 – 1.57) | 0.199 |
| **Need factors** | **Number of chronic conditions** | | - | - | - | - | 1.06 (0.98 – 1.15) | 0.175 |
|  | **Number of medications** | | - | - | - | - | 1.01 (0.96 – 1.06) | 0.734 |
|  | **Severity index (CIRS)** | | - | - | - | - | 1.01 (0.97 – 1.06) | 0.560 |
|  | **Burden of treatment** | | - | - | - | - | 1.10 (0.99 – 1.22) | 0.074 |
|  | **TBQ score** | | - | - | - | - | 1.00 (0.99 – 1.01) | 0.387 |
|  | **Mobility** | Not problematic | - | - | - | - | 1 (ref.) |  |
|  |  | Problematic | - | - | - | - | 1.27 (0.90 – 1.80) | 0.173 |
|  | **Autonomy** | Not problematic | - | - | - | - | 1 (ref.) |  |
|  |  | Problematic | - | - | - | - | 0.86 (0.52 – 1.42) | 0.548 |
|  | **Usual activities** | Not problematic | - | - | - | - | 1(ref.) |  |
|  |  | Problematic | - | - | - | - | 1.35 (0.93 – 1.94) | 0.112 |
|  | **Pain / Discomfort** | Not problematic | - | - | - | - | 1 (ref.) |  |
|  |  | Problematic | - | - | - | - | 2.49 (1.59 – 3.90) | **<0.001** |
|  | **Anxiety / Depression** | Not problematic | - | - | - | - | - | - |
|  |  | Problematic | - | - | - | - | - | - |
|  | **Health State (VAS)** | | - | - | - | - | 1.00 (0.99 – 1.01) | 0.355 |
|  | **Pseudo R^2^** | | **0.0116** |  | **0.0388** |  | **0.0889** |  |

1. Negative binomial regression for number of GP contacts in the last 12 months (Incidence-rate ratio (IRR) and 95% confidence interval (95% CI))

|  | | | Model 1 | | Model 2 |  | Model 3 | |
| --- | --- | --- | --- | --- | --- | --- | --- | --- |
|  |  |  | IRR (95% CI) | *p*-value | IRR (95% CI) | *p*-value | IRR (95% CI) | *p*-value |
| **Predisposing factors** | **Sex** | Men | 1 (ref.) |  | 1 (ref.) |  | 1 (ref.) |  |
|  |  | Women | 1.04 (0.95 – 1.13) | 0.386 | 1.04 (0.95 – 1.13) | 0.421 | 1.00 (0.92 – 1.08) | 0.974 |
|  | **Age** | <65 years | 1 (ref.) |  | 1 (ref.) |  | 1 (ref.) |  |
|  |  | 65-74 years | 1.05 (0.94 – 1.18) | 0.385 | 1.10 (0.98 – 1.24) | 0.107 | 1.11 (0.99 – 1.25) | 0.067 |
|  |  | 75-84 years | 1.20 (1.07 – 1.35) | **0.002** | 1.24 (1.10 – 1.40) | **<0.001** | 1.21 (1.07 – 1.36) | **0.002** |
|  |  | 85+ years | 1.17 (1.02 – 1.36) | **0.029** | 1.22 (1.05 – 1.42) | **0.009** | 1.26 (1.08 – 1.46) | **0.003** |
|  | **Martial state** | Married | 1 (ref.) |  | 1 (ref.) |  | 1 (ref.) |  |
|  |  | Single | 1.56 (1.33 – 1.83) | **<0.001** | 1.54 (1.32 – 1.81) | **<0.001** | 1.57 (1.35 – 1.82) | **<0.001** |
|  |  | Separated / Divorced | 1.18 (1.03 – 1.34) | **0.014** | 1.15 (1.01 – 1.31) | **0.033** | 1.12 (0.99 – 1.26) | 0.076 |
|  |  | Widow(er) | 1.11 (0.97 – 1.27) | 0.137 | 1.11 (0.97 – 1.27) | 0.137 | 1.08 (0.95 – 1.23) | 0.216 |
|  | **Number of adults in the household** | | 1.03 (0.94 – 1.12) | 0.498 | 1.04 (0.96 – 1.14) | 0.342 | 1.04 (0.96 – 1.12) | 0.389 |
|  | **Informal caregiver** | Presence | 1 (ref.) |  | 1 (ref.) |  | 1 (ref.) |  |
|  |  | Absence | 0.86 (0.79 – 0.93) | **<0.001** | 0.86 (0.79 – 0.94) | **0.001** | 0.90 (0.83 – 0.98) | **0.011** |
|  | **Schooling level** | Primary | 1 (ref.) |  | 1 (ref.) |  | 1 (ref.) |  |
|  |  | Secondary | 0.94 (0.85 – 1.05) | 0.289 | 0.96 (0.86 – 1.07) | 0.427 | 0.94 (0.85 – 1.04) | 0.220 |
|  |  | Tertiary | 0.86 (0.77 – 0.96) | **0.005** | 0.87 (0.78 – 0.97) | **0.012** | 0.86 (0.78 – 0.96) | **0.005** |
|  | **Linguistic region** | German | 1 (ref.) |  | 1 (ref.) | - | 1 (ref.) |  |
|  |  | French | 0.76 (0.70 – 0.83) | **<0.001** | 0.77 (0.71 – 0.83) | **<0.001** | 0.80 (0.73 – 0.86) | **<0.001** |
| **Enabling factors** | **Material Deprivation** | | - | - | - | - | - | - |
|  | **Social Deprivation** | | - | - | 0.98 (0.86 – 1.07) | 0.175 | 1.01 (0.98 – 1.04) | 0.543 |
|  | **Healthcare Deprivation** | | - | - | 1.09 (1.03 – 1.16) | **0.005** | 1.00 (0.93 – 1.07) | 0.928 |
| **Need factors** | **Number of chronic conditions** | | - | - | - | - | 0.99 (0.97 – 1.01) | 0.270 |
|  | **Number of medications** | | - | - | - | - | 1.03 (1.02 – 1.04) | **<0.001** |
|  | **Severity index (CIRS)** | | - | - | - | - | 1.01 (1.00 – 1.03) | **0.007** |
|  | **Burden of treatment** | | - | - | - | - | 1.08 (1.05 – 1.10) | **<0.001** |
|  | **TBQ score** | | - | - | - | - | 1.00 (1.00 – 1.00) | 0.511 |
|  | **Mobility** | Not problematic | - | - | - | - | 1 (ref.) |  |
|  |  | Problematic | - | - | - | - | 0.99 (0.91 – 1.08) | 0.786 |
|  | **Autonomy** | Not problematic | - | - | - | - | 1 (ref.) |  |
|  |  | Problematic | - | - | - | - | 0.87 (0.77 – 0.99) | **0.037** |
|  | **Usual activities** | Not problematic | - | - | - | - | 1(ref.) |  |
|  |  | Problematic | - | - | - | - | 1.09 (1.00 – 1.20) | **0.057** |
|  | **Pain / Discomfort** | Not problematic | - | - | - | - | - | - |
|  |  | Problematic | - | - | - | - | - | - |
|  | **Anxiety / Depression** | Not problematic | - | - | - | - | 1 (ref.) |  |
|  |  | Problematic | - | - | - | - | 1.04 (0.95 – 1.13) | 0.414 |
|  | **Health State (VAS)** | | - | - | - | - | 1.00 (1.00 – 1.00) | 0.254 |
|  | **Pseudo R^2^** | | **0.018** |  | **0.020** |  | **0.0428** |  |

1. Negative binomial regression for number of specialists (Incidence-rate ratio (IRR) and 95% confidence interval (95% CI))

|  | | | Model 1 | | Model 2 |  | Model 3 | |
| --- | --- | --- | --- | --- | --- | --- | --- | --- |
|  |  |  | IRR (95% CI) | *p*-value | IRR (95% CI) | *p*-value | IRR (95% CI) | *p*-value |
| **Predisposing factors** | **Sex** | Men | 1 (ref.) |  | 1 (ref.) |  | 1 (ref.) |  |
|  |  | Women | 0.99 (0.89 – 1.10) | 0.791 | 0.97 (0.87 – 1.07) | 0.515 | 0.93 (0.84 – 1.04) | 0.219 |
|  | **Age** | <65 years | 1 (ref.) |  | 1 (ref.) |  | 1 (ref.) |  |
|  |  | 65-74 years | 1.09 (0.94 – 1.26) | 0.272 | 1.21 (1.03 – 1.41) | **0.018** | 1.17 (1.00 – 1.37) | **0.050** |
|  |  | 75-84 years | 1.18 (1.02 – 1.36) | **0.026** | 1.34 (1.14 – 1.56) | **<0.001** | 1.19 (1.01 – 1.41) | **0.035** |
|  |  | 85+ years | 0.85 (0.70 – 1.03) | 0.101 | 1.00 (0.81 – 1.23) | 0.978 | 0.96 (0.77 – 1.20) | 0.728 |
|  | **Martial state** | Married | 1 (ref.) |  | 1 (ref.) |  | 1 (ref.) |  |
|  |  | Single | 0.86 (0.70– 1.07) | 0.172 | 0.87 (0.71 – 1.07) | 0.194 | 0.95 (0.77 – 1.17) | 0.601 |
|  |  | Separated / Divorced | 1.03 (0.87– 1.21) | 0.750 | 0.99 (0.84 – 1.17) | 0.950 | 1.03 (0.87 – 1.21) | 0.769 |
|  |  | Widow(er) | 0.85 (0.72 – 1.02) | 0.076 | 0.87 (0.73 – 1.03) | 0.112 | 0.88 (0.74 – 1.05) | 0.153 |
|  | **Number of adults in the household** | | 1.05 (0.95 – 1.18) | 0.337 | 1.07 (0.96 – 1.19) | 0.203 | 1.08 (0.97 – 1.20) | 0.167 |
|  | **Informal caregiver** | Presence | - | - | - | - | - | - |
|  |  | Absence | - | - | - | - | - | - |
|  | **Schooling level** | Primary | 1 (ref.) |  | 1 (ref.) |  | 1 (ref.) |  |
|  |  | Secondary | 1.09 (0.94 – 1.25) | 0.247 | 1.07 (0.93 – 1.23) | 0.354 | 1.07 (0.93 – 1.24) | 0.326 |
|  |  | Tertiary | 1.25 (1.08 – 1.44) | **0.002** | 1.21 (1.05 – 1.40) | **0.009** | 1.21 (1.05 – 1.40) | **0.008** |
|  | **Linguistic region** | German | 1 (ref.) |  | 1 (ref.) | - | 1 (ref.) |  |
|  |  | French | 1.14 (1.02 – 1.26) | **0.017** | 1.13 (1.02 – 1.26) | **0.018** | 1.23 (1.10 – 1.37) | **<0.001** |
|  | **Localization of GP’s office** | Urban | 1 (ref.) |  | 1 (ref.) |  | 1 (ref.) |  |
|  |  | Semi-urban | 0.91 (0.81 – 1.01) | 0.088 | 0.93 (0.83 – 1.03) | 0.165 | 0.88 (0.79 – 0.98) | **0.019** |
|  |  | Rural | 0.86 (0.74 – 1.00) | 0.053 | 0.88 (0.76 – 1.02) | 0.088 | 0.83 (0.72 – 0.97) | **0.016** |
| **Enabling factors** | **Material Deprivation** | | - | - | 1.03 (0.98 – 1.07) | 0.226 | 1.00 (0.96 – 1.05) | 0.993 |
|  | **Social Deprivation** | | - | - | 1.03 (0.99 – 1.07) | 0.105 | 1.07 (1.03 – 1.12) | **<0.001** |
|  | **Healthcare Deprivation** | | - | - | 1.18 (1.10 – 1.28) | **<0.001** | 1.02 (0.93 – 1.11) | 0.689 |
| **Need factors** | **Number of chronic conditions** | | - | - | - | - | 1.02 (1.00 – 1.05) | 0.081 |
|  | **Number of medications** | | - | - | - | - | 1.04 (1.02 – 1.06) | **<0.001** |
|  | **Severity index (CIRS)** | | - | - | - | - | 1.01 (1.00 – 1.03) | **0.041** |
|  | **Burden of treatment** | | - | - | - | - | 1.06 (1.02 – 1.10) | **0.001** |
|  | **TBQ score** | | - | - | - | - | 1.00 (1.00 – 1.00) | 0.405 |
|  | **Mobility** | Not problematic | - | - | - | - | 1 (ref.) |  |
|  |  | Problematic | - | - | - | - | 1.01 (0.90 – 1.13) | 0.908 |
|  | **Autonomy** | Not problematic | - | - | - | - | 1 (ref.) |  |
|  |  | Problematic | - | - | - | - | 0.95 (0.80 – 1.12) | 0.529 |
|  | **Usual activities** | Not problematic | - | - | - | - | 1(ref.) |  |
|  |  | Problematic | - | - | - | - | 1.05 (0.92 – 1.18) | 0.469 |
|  | **Pain / Discomfort** | Not problematic | - | - | - | - | 1 (ref.) |  |
|  |  | Problematic | - | - | - | - | 1.12 (0.98 – 1.28) | 0.107 |
|  | **Anxiety / Depression** | Not problematic | - | - | - | - | - | - |
|  |  | Problematic | - | - | - | - | - | - |
|  | **Health State (VAS)** | | - | - | - | - | 1.00 (1.00 – 1.00) | 0.789 |
|  | **Pseudo R^2^** | | **0.019** |  | **0.027** |  | **0.068** |  |

Model 1: controlled for predisposing factors

Model 2: controlled for predisposing factors (model 1) + enabling factors

Model 3: controlled for predisposing factors + enabling factors (model 2) + need factors
